# SiCNFe Ceramics as Soft Magnetic Material for MEMS Magnetic Devices: A Mössbauer Study

**DOI:** 10.3390/mi14050925

**Published:** 2023-04-25

**Authors:** Ion Stiharu, Sergey Andronenko, Almaz Zinnatullin, Farit Vagizov

**Affiliations:** 1 Department of Mechanical, Industrial and Aerospace Engineering, Concordia University, Montreal, QC H3G 1M8, Canada; 2 Institute of Physics, Kazan Federal University, 420008 Kazan, Russia; sergey.andronenko@gmail.com (S.A.); almaz.zinnatullin@gmail.com (A.Z.); vagizovf@gmail.com (F.V.)

**Keywords:** Mössbauer spectroscopy, SiCNFe composite, nanoparticles, MEMS magnetic actuators, soft magnets

## Abstract

Polymer-derived SiCNFe ceramics is a prospective material that can be used as soft magnets in MEMS magnetic applications. The optimal synthesis process and low-cost appropriate microfabrication should be developed for best result. Homogeneous and uniform magnetic material is required for developing such MEMS devices. Therefore, the knowledge of exact composition of SiCNFe ceramics is very important for the microfabrication of magnetic MEMS devices. The Mössbauer spectrum of SiCN ceramics, doped with Fe (III) ions, and annealed at 1100 °C, was investigated at room temperature to accurately establish the phase composition of Fe-containing magnetic nanoparticles, which were formed in this material at pyrolysis and which determine their magnetic properties. The analysis of Mössbauer data shows the formation of several Fe-containing magnetic nanoparticles in SiCN/Fe ceramics, such as α-Fe, Fe_x_Si_y_C_z_, traces of Fe-N and paramagnetic Fe^3+^ with octahedral oxygen environment. The presence of iron nitride and paramagnetic Fe^3+^ ions shows that the pyrolysis process was not completed in SiCNFe ceramics annealed at 1100 °C. These new observations confirm the formation of different Fe-containing nanoparticles with complex composition in SiCNFe ceramic composite.

## 1. Introduction

Various soft magnetic materials were developed for MEMS applications [1]. The possible use of soft and hard magnetic materials includes MEMS magnetic actuators, sensors, and micromotors. Soft magnetic materials are characterized by very low coercive fields and absence of magnetic hysteresis. Superparamagnetic compounds exhibit an absence of magnetic hysteresis along with rather large saturation magnetization and therefore can be used as soft magnets for various MEMS applications.

Polymer-derived SiCN ceramics is a prospective functional material for MEMS/NEMS applications [2,3,4,5,6,7,8,9]. Their magnetic and electrical properties can be adequately modified with transition metal ions doping. Therefore, such ceramics are of great interest in the development of high-temperature sensor applications [10,11,12], spintronic devices [13,14] and biological and medical applications [15,16]. SiCN ceramics can yield excellent mechanical properties and withstand very high temperatures. The microfabrication of different MEMS devices has been lately investigated [8,9].

SiCN is a polymer-derived ceramic whose starting material is a liquid-phase polymer. This provides it with an advantage of being molded into any desired shape. Thick and thin films of SiCN were made to suit the proposed application. CERASET™ also yields itself for photolithography with the addition of photo initiator 2,2-Dimethoxy-2-phenyl-acetophenone (DMPA), thus enabling photo lithographical patterning of the preceramic polymer using UV lithography. SiCN fabrication includes thermosetting, crosslinking and pyrolysis. This technology is still under investigation for enhanced stability and improved performance. In this respect, there are many engineering approaches that can solve the need for new aerospace materials and technologies that constantly address issues, such as viable product realization and maintenance, performance, costs, and environmental impact concerns. However, there are still many shortcomings affecting the implementation and integration of these approaches within the aerospace operations. This paper aims several objectives whose accomplishment would enable the development of MEMS/NEMS devices to be used in extremely harsh operating conditions, i.e., measurement of specific physical quantities at temperatures above 1000 °C [17,18].

The properties of SiCN ceramics can be modified by adding certain materials in the polymer. Thus, one can obtain, for example, magnetic materials with superparamagnetic properties which can be used as magnetic field sensors in very high temperatures. The magnetic impurities, e.g., Fe, Mn, Ni, Co or their compounds form nanoparticles inside SiCN ceramics with dimensions of 10–50 nm. Superparamagnetism then arises from the coupling of the magnetic moments of the individual transition metal ions within these nanoparticles. The so-called soft magnets, based on SiCN nanostructured ceramic samples possessing very low coercivity and negligible magnetic hysteresis, are the ones in which very large changes in the magnetic flux density can be produced by very small magnetic fields. Therefore, very sensitive magnetic sensors can be fabricated from SiCN ceramics.

The SiCN/Fe ceramic material was investigated intensively [19,20,21,22,23,24,25,26,27,28,29,30,31,32,33,34,35,36] and it was found that the magnetism of this material is a sum of different sources of magnetism: ferromagnetic nanoparticles with different compositions, superparamagnetic Fe^3+^ ion clusters and paramagnetic Fe^3+^ ions. Hence, one of very important aim to carefully control the chemical composition of SiCNFe ceramics with predictable magnetic properties. Therefore, the distribution of the nanoparticle sizes is preferred to be uniform and homogeneous and only the Fe-containing nanocrystallite is deemed desirable for best reproductivity.

SiCNFe ceramics also possess interesting electrical properties, e.g., they exhibit semiconducting behavior even at very high temperatures, as revealed by their conductivity [32].

Several investigations of SiCN doped with Fe ions were conducted recently [19,20,21,22,23,24,25,26,27,28,29,30,31,32,33,34,35,36]. These studies revealed that magnetic properties of SiCNFe composite are determined by the formation of magnetic nanoparticles, dispersed in diamagnetic SiCN nanoceramics. Chemical composition of these magnetic nanoparticles depends on the synthesis method, pyrolysis temperature, and the initial organic Si- and Fe-containing precursors. Numerous investigations show that Fe-containing nanoparticles are mainly α-Fe (T_C_ = 1043 K), Fe_3_Si (T_C_ = 390 K) and Fe_5_Si_3_ (T_C_ = 800 K), as it was reported in corresponding reviews [23,24,25,26]. However, the formation of Fe_2_SiO_4_ [19], Fe_3_C (T_C_ = 480 K) [20,21], Fe_3_N (T_C_ = 573 K), and Fe_4_N (T_C_ = 668 K) [22] phases was also observed. The objective of this work is a careful investigation of the phase composition of SiCN ceramics doped with Fe ions using the Mössbauer spectroscopy. Recently, this method was applied by Viard et al. [36] to SiCNFe fibers. They found that Mössbauer spectrum of solid polymer SiCNFe exhibits only doublet which corresponds to paramagnetic Fe^3+^ ions. The Mössbauer spectrum of SiCNFe ceramics (pyrolyzed at 1000 °C) with low level of Fe doping (0.1% Fe) has only one component: very broad singlet, ascribed to Fe_x_Si_y_C_z_ (without further clarification). The Mössbauer spectrum of the highly Fe-doped SiCNFe ceramics shows two components: a singlet attributed to α-Fe, and a sextet attributed to Fe_x_Si_y_ (without further refinement). Therefore, further Mössbauer studies of these ceramics, especially in the range of low iron content, are necessary for detailed understanding of Fe-containing phase composition.

## 2. Synthesis, Microfabrication and Characterization of SiCNFe Ceramics

### 2.1. SiCNFe Micromolding

**Figure 1:**
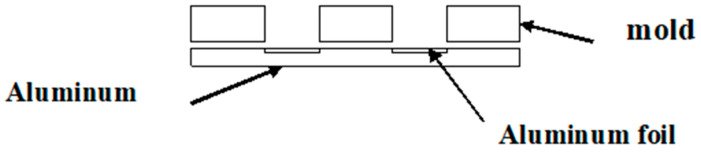
The aluminum mold for thermosetting liquid precursor.

Basic principles of polymer-derived SiCN micromolding were described by Li-Ann Lew et al. [2,3]. The chemistry of crosslinking and pyrolysis of CERASET™ was described by Ya-Li Li et al. [6]. SiCNFe micromolding is very similar to that of pure SiCN ceramics, but with some exceptions. In this study, the exception was the process of the formation of SiCNFe samples, which were under investigation. Liquid polymer precursor CERASET™ polyureasilazane, was doped with 5 wt.% of iron (III) acetylacetonate (fine powder) or iron (0) pentacarbonyl, or Fe(CO)_5_ (liquid). This solution was then stirred for two hours, and filtered to remove undissolved particles of powder to obtain a transparent dark-red liquid. First, a mold is fabricated using an 1 mm thick aluminum plate with several 4 mm diameter holes. The aluminum glue tape was glued from the bottom of this plate. The glue was isolated from the liquid precursor with very thin aluminum foil on the bottom of each hole. The aluminum mold is illustrated in Figure 1.

**Figure 2:**
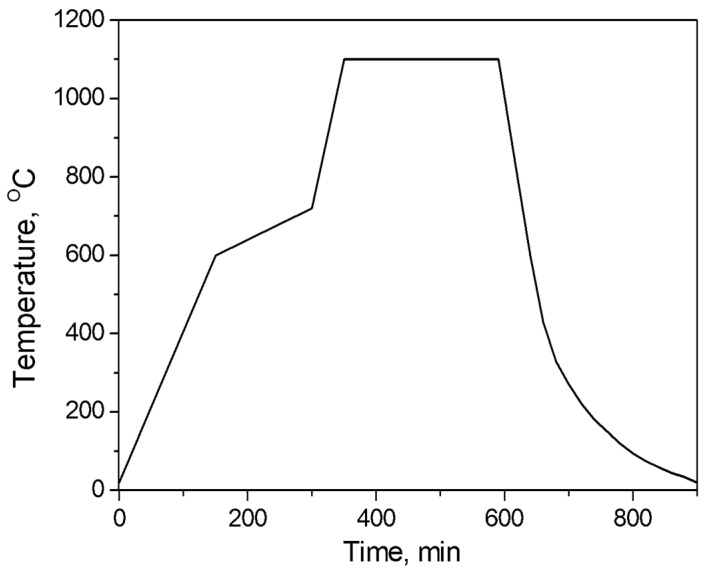
The dependence of treatment temperature of pyrolysis with time.

It is important to produce free-standing polymer structure in order to prevent stresses due to polymer shrinkage during crosslinking and pyrolysis. These stresses can completely destroy the ceramic structure at further heating. The liquid precursor is then cast into the mold, and the mold and CERASET™ polymer precursor are then heated or “thermal-set” at ~160 °C in a continuous flow of nitrogen gas for 60–90 min in order to solidify the polymer. After thermal-setting, the polymer becomes a gel-like dark-red solid, and may be separated from the mold by first removing the aluminum foil. The solid disks are placed on a silicon wafer and inserted into tube furnace once again. In the next step, the polymer discs are crosslinked by heating them to 400 °C in continuous nitrogen gas flow with the aid of the tube furnace for 90 min (heating rate is 4 °C/min and cooling rate is uncontrollable and it is approximately 2–5 °C/min). After crosslinking, the polymer becomes infusible, remaining transparent. In the final stage (pyrolysis), the crosslinked polymer wafer is heat-treated at 1100 °C to convert it to a monolithic ceramic material. This process is fulfilled in the same tube furnace (Barnstead Thermolyne 21135) in 4 h with continuous nitrogen gas flow. Heating rate is 4 °C/min from 20 °C to 600 °C, while from 600 °C to 720 °C, the heating rate is 1 °C/min. Higher heating rates results in the production of most of the gaseous by-products within a narrow temperature range (H_2_ at 600–700 °C, and CH_4_ at 600 °C), which could generate defects in the pyrolyzed samples and degrade the mechanical strength of the structures. Heating rate from 720 °C to 1000°C is 4 °C/min. Cooling rate is uncontrollable, starting from 15–20 °C/min (1000–800 °C) and decreasing to 10 °C/min (800–600 °C) and finally to 4–5 °C/min at lower temperatures. The dependence of treatment temperature of pyrolysis with time is shown in Figure 2.

Finally, we obtain hard ceramics discs which are 0.8 mm thick and 2.8 mm in diameter (shrinking is about 30%). However, majority of disks are usually destroyed during pyrolysis (during heating and cooling). Further improvement of the mechanical properties of SiCN ceramics can be performed using an isostatic pressure process.

**Figure 3:**
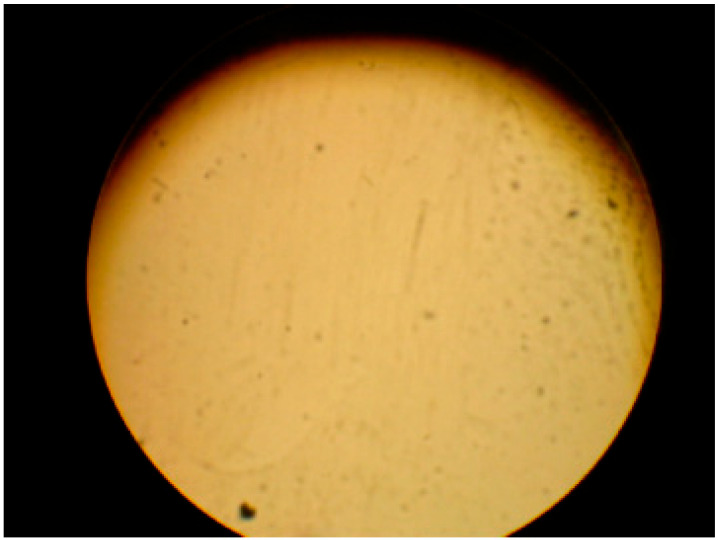
Surface of SiCN ceramic disk under 100 times magnification.

As shown in Figure 3, no cracks were seen on the surface. Temperature treatment is 1100 °C for pyrolysis with slow heating rate between 600 °C and 720 °C.

### 2.2. SiCN Films Fabrication with Use of UV Lithography

#### 2.2.1. Optimal Composition of Liquid Polymer and Possible Substrates

The synthesis of SiCN ceramics from polyureasylazane (CERASET™) was previously described in detail [2,3,4,5]. Initial polyureasylazane–iron (III) acetylacetonate solution was prepared as described in Section 2. The liquid precursor requires three characteristics in order to make successful ceramic coating: (1) Turn into SiCN upon heat treatment, (2) Sensitive to light energy and polymerize upon exposure, (3) Wet the surface of the chosen substrate. Hence, the liquid precursor consists of three materials each contributing to one of the above needs. Commercially available liquid silazane, CERASET™, is the core material of the precursor which forms SiCN upon heat treatment. CERASET™ is diluted with acetonitrile to enhance wetting and then mixed with 2,2-Dimethoxy-2-phenyl-acetophenone (DMPA), a type of photo-initiator. CERASET™ is a viscous liquid, which in its undiluted form does not wet substrate surfaces well. DMPA, the photo-curing agent, initiates the polymerization reaction in the CERASET™ by producing a radical monomer. The DMPA content in the mixture determines the curing rate. The optimum precursor composition by weight is CERASET™:Acetonitrile:DMPA = 65%:30%:5%. Substrates used for the fabrication are Si, SiC and Zirconia. We used wafer Si (100) 3,5 inches. Substrate plays an important role since samples are heat-treated to high temperatures, and being at such a high temperature causes diffusion between the amorphous SiCN and the substrate. The substrate also affects the resolution of the micro-patterning. For instance, the resolution of the microstructure on a one side polished sapphire substrate is poor whereas the silicon substrate promotes excellent resolution. Silicon substrates possess a superior optical property which does not diffract the incident light energy at all. Silicon substrates can be used, however, only to moderate temperatures because of its low melting point (1410 °C). Yttria partially stabilized zirconia has 1500 °C maximum operating temperature and the unpolished side of the zirconia substrate is used for sensor fabrication. Other candidates for substrates are sapphire, Si_3_N_4_ (Silicon Nitride) and quartz.

#### 2.2.2. Spin-Coating

Because of its high viscosity, the polysilazane precursor solution is spin-coated at 6100 rpm for 30 s. Covering polymer is conducted using spin-coater Laurell (Lansdale, PA, USA) WS-400 6100 rpm acceleration-500 rpm/s. This setting provides good uniformity and thickness. Unpolished (lapped) side of the substrate is used for better adhesion of the SiCN onto the substrate. The polysilazane mixture acts like a negative photoresist and cures on the exposed areas. When the polymer microstructure is too thin, it disintegrates and disappears during heat treatment process. Some form of surface chemistry takes place which deteriorates the coating. If the structure is too thick, the structure cracks after the heat treatment due to the thermal mismatch with the substrate. Due to the irregular surface of the lapped substrate, measurement of the critical thickness is not possible.

#### 2.2.3. Heat Treatment

Heat treatment consists of densification of the gel-like polymer into an infusible solid structure, and pyrolysis. These two heat treatment processes take place in a nitrogen environment. When the liquid precursor is cured under UV light, it is polymerized into a gel-like structure which is still soluble in organic solvents. It is further thermally crosslinked at 400 °C into an infusible solid (densification) before being converted into an amorphous SiCN ceramic by pyrolysis. During the densification process, the unpolymerized oligomer molecules are further crosslinked into large molecules with highly interlocked backbones. Without the densification, the oligomers can evaporate from the structure during the pyrolysis process and cause a porous SiCN coating. The pyrolysis process takes place at 1100 °C in the Furnace Tube Barnstead Thermolyne 21135 (Clarkson Laboratory, Chula Vista, CA, USA). During pyrolysis, the polymer decomposes into an amorphous SiCN structure with gases, such as methane, slowly evolving from the structure. At this stage, the amorphous SiCN is non-conducting. Densification occurs at 400 °C for 1.5 h and pyrolysis occurs at 1100 °C for 3 h. All three heat treatments occur in a tube furnace with continuous nitrogen flow. Heating rates are 5 °C/min.

**Figure 4:**
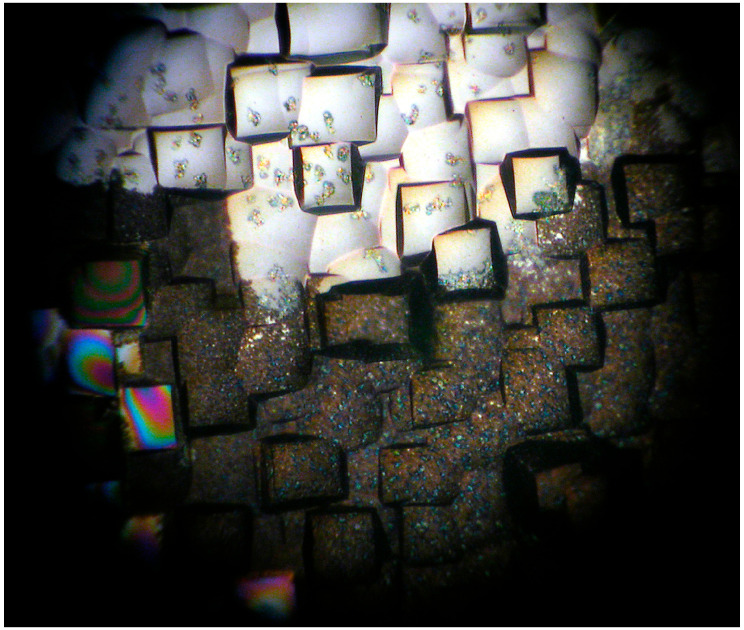
The surface after pyrolysis on Si (100) substrate (with spin-coating at 5000 rpm). The magnification is 100 times.

**Figure 5:**
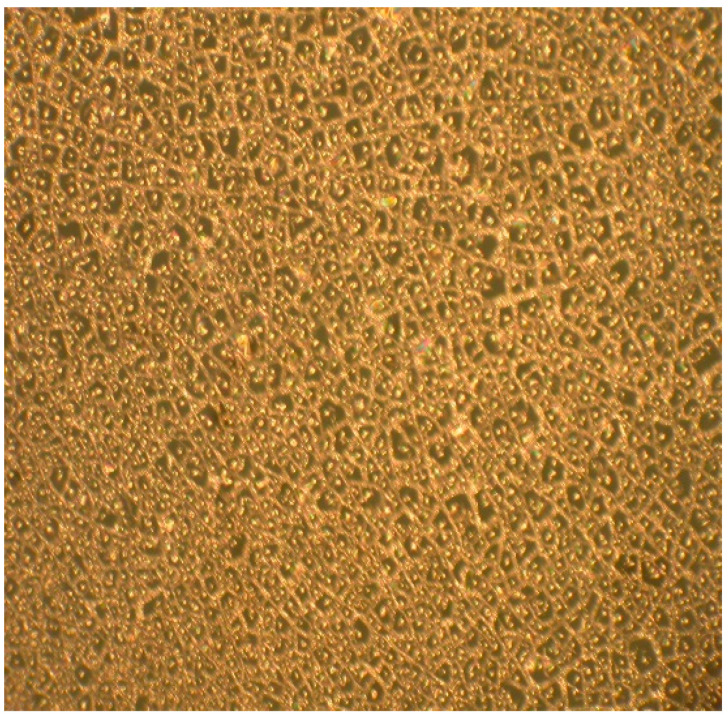
The surface of SiCN films on ZrO_2_ substrate after pyrolysis (spin-coating at 6100 rpm and ZrO_2_ substrate, unpolished). There are no visible cracks on the surface. The magnification is 100 times.

It can be seen from Figure 4 that there are evident cracks on the surface, because of the increased thickness of the coating. We could obtain the best results only for high-speed coating (at 6100 rpm, maximum speed of spin-coating machine and crystalline ZrO_2_ substrate (100) plane, unpolished), as shown in Figure 5.

### 2.3. SEM Image of SiCNFe Sample Annealed at 1100 °C

**Figure 6:**
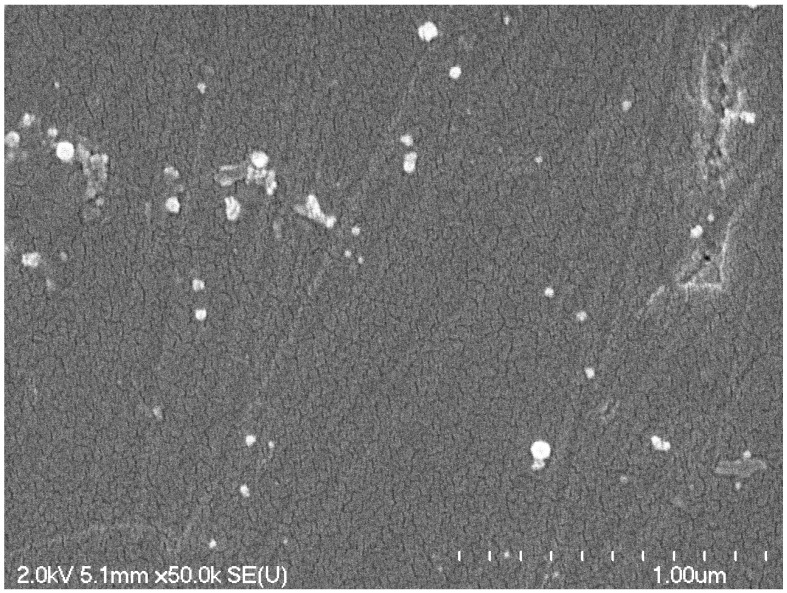
SEM image of SiCN ceramics annealed at 1100 °C.

The samples of SiCNFe annealed at 1100 °C are still amorphous, as it was found by XRD studies [27]. The crystallization of SiCNFe ceramics starts from annealing temperature ~1200 °C, as it can be seen from XRD patterns [27]. The average dimensions of nanocrystallites of SiCN ceramics were determined by Leo et al. [11] from SEM image as 40 nm for SiCN sample annealing at 1500 °C. The SEM image of the sample, annealed at 1100 °C, also revealed its amorphous microstructure with several nanocrystalline inclusions. There are no visible grain boundaries. Additional inclusions have average dimensions less than 50 nm, as it can be seen in Figure 6. The chemical composition of these inclusions is unknown. It is supposed that these crystallites are mainly α-Fe, and Fe_5_Si_3_, as determined by Andronenko et al. [27]. However, it is quite possible that this sample contains other nanocrystallites with other chemical composition.

### 2.4. Magnetic Properties of SiCNFe Annealed at 1100 °C

**Figure 7:**
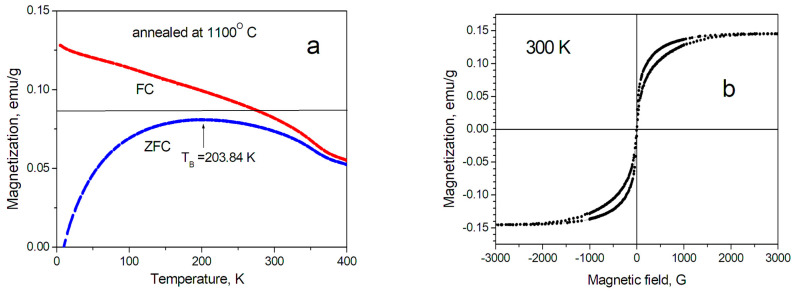
Magnetic properties of SiCNFe ceramics annealed at 1100 °C [28]. (**a**) Temperature dependences of the ZFC (zero field cooling) and FC (field cooling) magnetizations for the SiCN/Fe. (**b**) Magnetic field dependence of the magnetization at 300 K.

The magnetic properties of SiCNFe samples, annealed at various temperatures, from 600 to 1485 °C, were investigated and analyzed by Andronenko et al. [28]. However, it is worth to repeat some useful properties of SiCNFe sample, annealed at 1100 °C. The composition of these polymer-derived ceramics, as determined by elementary chemical analysis, was found to be SiC_0.68_N_0.41_. Neutron-activated analysis of SiCN/Fe composites annealed at 1100 °C determined the Fe content to be 0.2 wt.% [27]. The investigations of the SiCNFe magnetization clearly show that Fe-containing inclusions also exhibit nanostructures [28]. Blocking temperature was found to be 204 K (Figure 7a) for SiCNFe sample annealed at 1100 °C. As it can be seen in Figure 7b, hysteresis was not observed for this sample and coercive field is really zero. However, noticeable coercive field appears for SiCNFe samples annealed at lower temperature. For example, for SiCNFe sample annealed at 1000 °C, the coercive field was found to be 74 G [28]. Therefore, annealing temperature should be not less than 1100 °C. At the same time, as described above, SiCNFe samples, annealed at 1100 °C, can be obtained in a rather economic furnace, Barnstead Thermolyne 21135, with a highest temperature of 1200 °C. Hence, this pyrolysis process is not expensive and provides really soft magnet material. Therefore, the investigation of SiCNFe samples annealed at 1100 °C is of interest for soft magnet material development.

## 3. Mössbauer Spectrum of SiCNFe Ceramics Annealed at 1100 °C

### 3.1. The Instrumentation and Analysis

Mössbauer spectrum of the sample under study was recorded on the conventional WissEl spectrometer working in the constant acceleration mode. Measurement was carried out in transmission geometry. ^57^Co(Rh) source with an activity of about 40 mCi was used. The spectrometer velocity scale was calibrated using the spectrum of a thin bcc-Fe foil. The raw spectrum was processed using SpectrRelax 2.1 software [37]. The center shift values versus gravity center of bcc-Fe spectrum at the room temperature are presented.

Room temperature Mössbauer spectrum of the sample SiNCFe is shown in Figure 7. The spectrum was least square fitted within the model consisting of four components. The obtained hyperfine parameters of these components are listed in Table 1.

### 3.2. The Interpretation of Mössbauer Spectrum

#### 3.2.1. Component I

**Figure 8:**
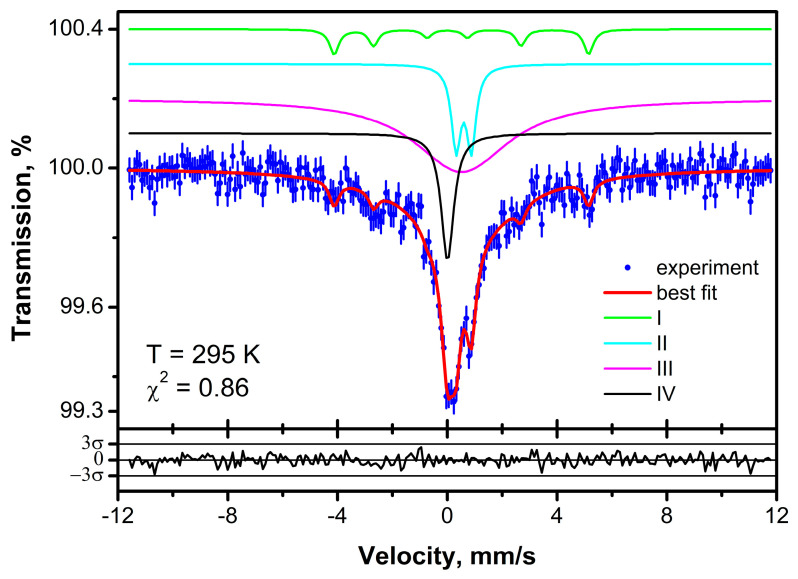
Room temperature Mössbauer spectrum of the sample SiNCFe annealed at 1100 °C. Blue points represent experimental spectrum with statistical error bars. Red curve is the best-fit result. The model components are shown by colored lines. Residuals between experimental spectrum and fitting curve (expressed in units of statistical error) are plotted in the bottom.

Certainly, the revealed sextet in Mössbauer spectrum (component I, light green curve in Figure 8) is associated with magnetically ordered iron atoms. According to the literature data, the hyperfine parameters of this sextet are closer to the values for the compounds with the nominal composition of Fe*_x_*Si*_y_*C*_z_* with larger content of iron [34,35], than for other systems with combinations of current chemical elements (Fe, Si, C, N). The formation of nanoparticles of Fe_5_Si_3_ and Fe_3_Si was observed in several SiCNFe ceramics compounds synthesized using numerous different methods [23,24,25,26]. For example, the formation of Fe_5_Si_3_ was detected using the EPR method in SiCNFe ceramics annealed at 1100 °C [27]. The mathematical processing of temperature dependence of EPR linewidth and EPR integral intensity provides the value of Curie temperature, 390 K, which exactly corresponds to the magnetic phase transition of Fe_5_Si_3_ [39].

#### 3.2.2. Component II

The doublet, which is shown by the cyan curve in Figure 8 (component II), has hyperfine parameters uncharacteristic for the compounds, containing the combination of the main chemical elements (Fe, Si, C, and N). Similar values of isomer shifts and quadrupole splittings are typical for high-spin ferric ions in oxygen octahedral environment [40]. Therefore, based on Mössbauer results, we suppose that this doublet is related to paramagnetic [Fe^3+^O_6_] centers. This is supported by the EPR investigations of solid polymers crosslinked at 400 °C, and SiCNFe preceramics annealed at 600–900 °C. The narrow EPR spectra with g_1_ = 4.3 and g_2_ = 2.04 were observed in these compounds [27]. These signals correspond to the paramagnetic state of the Fe^3+^ ion. The Mössbauer spectrum of solid polymer SiCNFe [36] exhibit doublet which also ascribed to paramagnetic Fe^3+^. That proves a formation of paramagnetic state of Fe^3+^ ions in SiCNFe ceramics, annealed at 1100 °C. Furthermore, the oxygen ions still exist in SiCN annealed at 1100 °C, as it was revealed from ^13^C and ^29^Si NMR spectra [10]. Therefore, the formation of the Fe^3+^-related paramagnetic centers is possible for SiCNFe ceramics where pyrolysis process was not fully completed. Fainer et al. [33] also observed highly anisotropic EPR signal of paramagnetic centers in SiCNFe films with low Fe concentration annealed at 300–600 °C. They supposed that these centers are [Fe(CN)_6_]^3−^ ions. However, in such centers, iron ions should be in the low-spin state like it was reported for K_3_Fe(CN)_6_ [41]. The low-spin iron ions are characterized by the lower values of isomer shift [39]. However, we do not observe such component in our spectrum. Thus, the formation of [Fe(CN)_6_]^3−^ in our sample seems to be unlikely. At the same time, Fainer et al. [26] observed very broad EPR spectrum with g_1_ = 4.3 and g_2_ = 4.15 in SiCNFe films with very high Fe concentration. This spectrum can correspond to ferrimagnetic Fe_2_O_3_ [42]. As mentioned above, the currently investigated SiCNFe sample with very low Fe concentration exhibits the EPR spectrum with similar g values, but with low linewidth. Therefore, we can suppose that this spectrum corresponds to the clusters of Fe^3+^-O_6_, which form magnetically ordered compound as Fe concentration increase.

#### 3.2.3. Component III

Component III has a broadened lineshape with the center at 0.5(1) mm/s. This component is depicted by the magenta-colored curve in Figure 8. Similar values of isomer shifts are typical for iron nitrides FeN*_x_* with *x* < 0.5 [38]. Possible reason for broadening of this component is a distribution of hyperfine parameters due to the dispersion of *x* values. Furthermore, for *x* < 0.4, FeN*_x_* were found in the magnetically ordered state at room temperature, and the hyperfine magnetic field magnitudes depend on *x* value as well [38]. The formation of iron nitride, Fe_4_N, and Fe_3_N_,_ was only reported by Li et al. [22] for SiCNFe samples annealed at 900 °C in NH_3_ gas atmosphere. Therefore, it is possible that such ferromagnetic iron nitrides still exist in SiCNFe ceramics annealed at 1100 °C.

#### 3.2.4. Component IV

The singlet component also appears in the spectrum (component IV, black curve in Figure 8). It has an isomer shift of 0.00(3) mm/s relative to metallic iron at room temperature. Based on this observed value and the absence of quadrupole and magnetic splittings, we suppose that this component is related to small particle/clusters of α-Fe. The magnetic hyperfine structure of bcc-Fe may not be observed at room temperature due to superparamagnetic relaxation resulting in the collapse magnetically ordered pattern (sextet) into a single absorption line [43]. The formation of α-Fe nanoparticles in SiCNFe ceramics was reported in numerous publications [23,24,25,26,27,28,29,30,31,32,33,36] and this is expected for SiCNFe annealed at 1100 °C. The EPR spectra of these samples belonging to α-Fe nanoparticles were discussed by Misra et al. [28] and splitting of the W-band EPR spectra were observed [29]. Clear evidence of the formation of both α-Fe nanoparticles and large α-Fe clusters was obtained recently by Stepina et al. [32] in granulated SiCNFe films with high Fe concentration.

## 4. Conclusions

In the recent work [36], similar Mössbauer spectrum for the sample of SiCNFe fibers with low concentration of iron and pyrolysis temperature of 1000 °C have been reported. However, authors processed the raw spectrum within single broad absorption line centering at 0.33 mm/s and attributed this component to iron silicide phases. Our results show that the nature of iron containing silicon nitrocarbides is more complex. Iron ions were found in the various valence and magnetic states. The formed composite structure determines peculiarities in the magnetic properties of these ceramics.

The formation of α-Fe, Fe_5_Si_3_, and Fe_3_Si nanoparticles is usual for SiCNFe ceramics annealed at 1100 °C. However, the presence of the traces of iron nitrides of paramagnetic Fe^3+^ ions were reported only for SiCNFe preceramics annealed below 900 °C. The Mössbauer spectrum shows that their presence is quite possible. Therefore, we should suppose that pyrolysis process was not fully completed in SiCNFe ceramics annealed at 1100 °C. As it was found by Andronenko et al. [10], hydrogen ions are completely removed from SiCN ceramics only at 1150 °C and this could be a sign for complete transformation of polymer precursor to solid ceramics. Therefore, some traces of iron nitride or paramagnetic Fe^3+^ ions can be detected in the SiCNFe samples annealed below 1150 °C.

Finally, we concluded that optimal annealing temperature for synthesis of SiCNFe ceramics should be not less than 1200 °C.

The most important result of the present Mössbauer studies on SiCN/Fe ceramic samples annealed at 1100 °C are as follows:

1. The rigorous analysis of Mössbauer spectrum of the SiCNFe sample annealed at temperature 1100 °C revealed the presence of α-Fe, Fe_5_Si_3_ nanoparticles and, probably, Fe_3_Si nanoparticles also. However, there are also the traces of iron nitrides (Fe_3_N, and Fe_4_N) and Fe^3+^ ions in octahedral oxygen environment.
2. The presence of iron nitride and paramagnetic Fe^3+^ ions shows that pyrolysis process was not completed in SiCNFe ceramics annealed at 1100 °C.
3. The best results for SiCNFe films on the substrate were obtained only for high-speed coating (at 6100 rpm, maximum speed of spin-coating machine and crystalline ZrO_2_ substrate (100) plane, unpolished).

## Figures and Tables

**Table 1:** Hyperfine parameters of room temperature Mössbauer spectrum components for the sample SiNCFe.

Component	CS	QS	HF	W	A	Assigned Phase
I	0.26(4)	0.52(8)	288(3)	0.4(1)	9(3)	Fe rich Fe_x_Si_y_C_z_ [34,35]
II	0.60(3)	0.56(4)	-	0.42(8)	14(3)	[Fe^3+^O_6_]
III	0.5(1)	-	-	4.2(8)	63(4)	FeN_x_ with distribution x < 0.5 [38]
IV	0.00(3)	-	-	0.55(8)	15(2)	Superparamagnetic α-Fe

CS—center shift in mm/s relative α-Fe at room temperature; QS—quadrupole splitting in mm/s; HF—hyperfine magnetic field on ^57^Fe nuclei in kOe; W—the linewidth in mm/s; A—relative area of the component in %.

## Data Availability

Not applicable.

## References

[1] Wu S., Hu W., Ze Q., Sitti M., Zhao R. (2020). Multifunctional magnetic soft composites: A review. Multifunct. Mater..

[2] Liew L.-A., Zhang W., Bright V.M., An L., Dunn M.L., Raj R. (2001). Fabrication of SiCN ceramics MEMS using injectable polymer-precursor technique. Sens. Actuators A Phys..

[3] Liew L.-A., Liu Y., Luo R., Cross T., An L., Bright V.M., Dunn M.L.L., Daily J.W., Raj R. (2002). Fabrication of SiCN MEMS by photopolymerization of pre-ceramic polymer. Sens. Actuators A Phys..

[4] Bright V.M., Raj R., Dunn M.L., Daily J.W. (2004). Injectable Ceramic Microcast Silicon Carbon Nitride (SiCN), Micro Electro Mechanical Systems (MEMS) for Extreme Temperature Environments with Extension: Micro Packages for Nano Devices.

[5] Liew L.-A., Saravanan R.A., Bright V.M., Dunn M.L., Daily J.W., Raj R. (2003). Processing and caracterization of silicon carbon-nitride ceramics: Application of electrical properties toward MEMS thermal actuators. Sens. Actuators A Phys..

[6] Li Y.-L., Riedel R., Steiger J., von Seggem H. (2000). Novel Transparent Polysilazane Glass: Synthesis and Properties. Adv. Eng. Mater..

[7] Schulz M. (2009). Polymer Derived Ceramics in MEMS/NEMS—A Review on Production Processes and Application. Adv. Appl. Ceram..

[8] Xia X., Yin J., Chen X., Liu X., Huang Z. (2020). Polymer-Derived Si-Based Ceramics: Recent Developments and Perspectives. Crystals.

[9] Hagelüken L., Sasikumar P.V.W., Lee H.-Y., Di Stadio D., Chandorkar Y., Rottmar M., Maniura-Weber K., Blugan G., Brugger J. (2022). Multiscale 2D/3D microshaping and property tuning of polymer-derived SiCN ceramics. J. Eur. Ceram. Soc..

[10] Andronenko S.I., Stiharu I., Misra S.K. (2006). Synthesis and characterization of polyureasilazane derived SiCN ceramics. J. Appl. Phys..

[11] Leo A., Andronenko S., Stiharu I., Bhat R.B. (2010). Thick and thin film SiCN for pressure sensor at high temperature and influence of temperature on mechanical properties. Sensors.

[12] Leo A., Andronenko S.I., Stiharu I. (2018). Fabrication and machining of SiCN for pressure sensor at high temperature for aero-engines applications. Int. J. Mater. Sci. Res..

[13] Fujimori H., Ohnuma S., Kobayashi N., Masumoto T. (2006). Spintronics in metal-insulator nanogranular magnetic thin films. J. Magn. Magn. Mater..

[14] Wolf S.A., Awschalom D.D., Buhrman R.A., Daughton J.M., von Molnár S., Roukes M.L., Chtchelkanova A.Y., Treger D.M. (2001). Spintronics: A spin-based electronics vision for the future. Science.

[15] Chan M.-H., Li C.-H., Chang Y.-C., Hsiao M. (2022). Iron-Based Ceramic Composite Nanomaterials for Magnetic Fluid Hyperthermia and Drug Delivery. Pharmaceutics.

[16] Bao Y.P., Wen T.L., Samia A.C.S., Khandhar A., Krishnan K.M. (2016). Magnetic nanoparticles: Material engineering and emerging applications in lithography and biomedicine. J. Mater. Sci..

[17] Ren Z., Mujib S.B., Singh G. (2021). High-Temperature Properties and Applications of Si-Based Polymer-Derived Ceramics: A Review. Materials.

[18] Zhao R., Shao G., Cao Y., An L., Xu C. (2014). Temperature sensor made of polymer-derived ceramics for high-temperature applications. Sens. Actuators A Phys..

[19] Saha A., Shah S.R., Raj R., Russek S.E. (2003). Polymer derived SiCN composite with magnetic properties. J. Mater. Res..

[20] Dumitru A., Ciupina V., Stamatin I., Prodan G., Morozan A., Mirea C. (2006). Plasma polymerization of ferrocene with silane and silazane monomers for design of nanostructured magnetic ceramics. J. Optoelectron. Adv. Mater..

[21] Dumitru A., Stamatin I., Morozan A., Mirea C., Ciupina V. (2007). Si-C-N-Fe nanostructured ceramics from inorganic polymer precursors obtained by plasma polymerization. Mater. Sci. Eng. C.

[22] Li Y., Zheng Z., Reng C., Zhang Z., Gao W., Yang S., Xie Z. (2003). Preparation of Si-C-N-Fe magnetic ceramics from iron-containing polysilazane. Appl. Organomet. Chem..

[23] Hojamberdiev M., Prasad R.M., Fasel C., Riedel R., Ionescu E. (2013). Single-source-precursor synthesis of soft magnetic Fe_3_Si- and Fe_5_Si_3_-containing SiOC ceramic nanocomposites. Eur. J. Ceram. Soc..

[24] Zaheer M., Schmalz T., Motz G., Kempe R. (2012). Polymer derived non-oxide ceramics modified with late transition metals. Chem. Soc. Rev..

[25] Mera G., Gallei M., Bernard S., Ionescu E. (2015). Ceramic nanocomposites from tailor-made preceramics polymers. Nanomaterials.

[26] Yan X., Cheng X., Han G., Hauser R., Riedel R. (2007). Synthesis and magnetic properties of polymer derived metal/SiCN ceramic composites. Key Eng. Mater..

[27] Andronenko S.I., Stiharu I., Misra S.K., Lacroix C., Menard D. (2010). EPR/FMR, FTIR, X-ray, and Raman investigations of Fe-doped SiCN ceramics. Appl. Magn. Reson..

[28] Misra S.K., Andronenko S., Gilmutdinov I., Yusupov R. (2018). EPR and magnetization studies of polymer-derived Fe-doped SiCN nanoceramics annealed at various temperatures: Blocking temperatures, superparamagnetism and size distribution. Appl. Magn. Reson..

[29] Andronenko S.I., Rodionov A.A., Misra S.K. (2018). A variable temperature X- and W-band EPR study of Fe-doped SiCN ceramics annealed at 1000 °C, 1100 °C, and 1285 °C: Dangling bonds, ferromagnetism and superparamagnetism. Appl. Magn. Reson..

[30] Pushkarev R.V., Fainer N.I., Maurya K.K. (2017). Structural and magnetic properties of nanocomposite iron-containing SiC_x_N_y_ films. Superlattices Microstruct..

[31] Pushkarev R., Fainer N., Kirienko V., Matsynin A., Nadolinnyy V., Merenkov I., Trubina S., Ehrenburg S., Kvashnina K. (2019). SiC_x_N_y_:Fe films as a tunable ferromagnetic material with tailored conductivity. J. Mater. Chem. C.

[32] Stepina N.P., Pushkarev R.V., Zinovieva A.F., Kirienko V.V., Bogomyakov A.S., Gutakovskii A.K., Fainer N.I., Dvurechenskii A.V. (2020). Magnetic properties of granulated SiC_x_N_y_:Fe films with different structure of α-Fe nanoclusters. J. Magn. Magn. Mater..

[33] Fainer N.I., Plekhanov A.G., Pushkarev R.V., Shayapov V.R., Maksimovskiy E.A., Nadolinny V.A., Korotaev E.V., Kaichev V.V. (2020). Synthesis of magnetic nanocomposite films SiCxNyFez by plazma-enhanced chemical decomposition of a gaseous mixture of 1,1,1,3,3,3-hexamethyldisilazane, ferrocene, and helium. J. Struct. Chem..

[34] Lomayeva S.F., Maratkanova A.N. (2009). Structure, phase composition and magnetic properties of Fe-Si-C system due to mechanical activation of Fe-Si alloy in organic liquids. Intermetallics.

[35] Yelsukov E.P., Maratkanova A.N., Lomayeva S.F., Konyagin G.N., Nemtsova O.M., Ul’yanov A.I., Chulkina A.A. (2006). Structure, phase composition and magnetic properties of mechanically alloyed and annealing quasibinary Fe(70)Si(x)C(30-x) alloys. J. Alloys Compd..

[36] Viard A., Kurz H., Lale A., Heymann L., Weber B., Bernard S., Knauer M., Motz G. (2021). Superparamagnetic silicon carbonitride ceramic fibers through in situ generation of iron silicide nanoparticles during pyrolysis of an iron-modified polysilazane. ACS Appl. Mater. Interfaces.

[37] Matsnev M.E., Rusakov V.S. (2012). SpectrRelax: An application for Mössbauer spectra modeling and fitting. AIP Conf. Proc..

[38] Mekata M., Yoshimura H., Takaki H. (1972). Magnetic study on hexagonal nitrides of 3*d* transition metals. J. Phys. Soc. Jpn..

[39] Sawatzky E. (1971). Magnetic and magnetooptical properties of sputtered Fe_5_Si_3_ films. IEEE Trans. Magn..

[40] Murad E., Cashion J. (2014). Mossbauer Spectroscopy of Environmental Materials and Their Industrial Utilization.

[41] Oosterhuis W.T., Lang G., Debenedetti S. (1967). Mössbauer spectra of K_3_Fe(CN)_6_ at low temperatures. Phys. Lett. A.

[42] Ren X., Zhang W., Zhang Y., Zhang P., Liu J. (2015). Effects of Fe_2_O_3_ content on microstructure and mechanical properties of CaO-Al_2_O_3_-SiO_2_ system. Trans. Nonferrous Met. Soc. China.

[43] Afanas’ev A.M., Chuev M.A. (2001). New relaxation model for superparamagnetic particles in Mössbauer spectroscopy. J. Exp. Theor. Phys. Lett..

